# Genome-wide identification of peanut IGT family genes and their potential roles in the development of plant architecture

**DOI:** 10.1038/s41598-023-47722-4

**Published:** 2023-11-21

**Authors:** Wen Chu, Xiaofeng Zhu, Tao Jiang, Song Wang, Wanli Ni

**Affiliations:** Crops Research Institute, Anhui Academy of Agricultural Sciences, Hefei, 230031 China

**Keywords:** Evolution, Genetics

## Abstract

IGT family genes play essential roles in shaping plant architecture. However, limited amount of information is available about IGT family genes in peanuts (*Arachis hypogaea*). In the current study, 13 *AhIGT* genes were identified and classified into three groups based on their phylogenetic relationship. Gene structure, conserved domain analyses indicated all *AhIGTs* were observed to share a similar exon–intron distribution pattern. AhIGTs within the same subfamily maintained a consistent motif composition. Chromosomal localization and synteny analyses showed that *AhIGTs* were unevenly localized on 9 chromosomes and that segmental duplication and purifying selection may have played important roles in the evolution of *AhIGT* genes. The analysis of conserved motifs, GO annotation, and transcript profile suggested that *AhLAZY1-3* may play roles in gravity sensing and shaping peanut plant architecture. Transcript profile analysis suggested that *AhTAC1* could potentially be involved gynophore (‘peg’) penetration into the soil. The *cis*-element analysis revealed that the light-responsive elements accounted for most of all *cis*-acting elements. Furthermore, qRT-PCR analysis showed that the expression of several *AhIGT* genes, like *AhTAC1-2/4*, was light-dependent, indicating that these genes may regulate plant architecture in response to light signals. This study may facilitate functional studies of the *IGT* genes in peanut.

## Introduction

Optimizing plant architecture has been demonstrated as one of the best methods to improve planting density, stress-tolerance and overall productivity of crops^1^. The IGT gene family, which is identified based on a conserved motif (GφL(A/T)IGT), has been observed to play a significant role in regulating gravitropism and shaping the structure of plants^2^. The IGT gene family comprises a set of genes (*LAZY1-6*) and *TILLER ANGLE CONTROL 1* (*TAC1*). *IGT* genes have relatively low levels of sequence conservation across gene-family members. Nevertheless, 5 short conserved motifs, called I–V domains, have been identified in IGT family proteins, but not all were present in all members of the family^3^. Additionally, a unique intron–exon arrangement was also detected for *IGT* genes. This arrangement was characterized by a short exon encoding two amino acids and the last exon encoding a short peptide including ethylene-responsive amphiphilic repression (EAR) motif containing transcriptional repressors^3,4^. TAC1 lacks this motif (domain V) which is known to be essential for functions of LAZY1 and LAZY4^5^. Domain I located at N terminus, and it is necessary for anchoring AtLAZY1 at the plasma membrane^6^. The IGT conserved domain II contains the family-specific GφL (A/T) IGT sequence. Any mutation in this directly resulted in the loss of function of LAZY1^4,6^. Much less information about the domain III and IV is available^7–9^.

The *lazy1* traits, initially described in the 1930s, exhibited a prostrate growth habit in rice and maize^10,11^. Over the decades, the *LAZY1*, which responsible for *lazy* traits^12–14^ have been identified. *LAZY1* is a crucial regulator of negative gravitropism, and its loss leads to the formation of wide-angled branches in *Arabidopsis thaliana*^15^. *LAZY4*/*DEEPER ROOTING 1* (*DRO1*) was identified as a key QTL controlling deep rooting in rice^16^. LAZY4 is involved in gravitropic responses via auxin signaling and promotes deep rooting in rice^17^. *TAC1* was described as a key QTL that controlled the production of compact and erect tillers in rice^18^. *TAC1* showed a similar expression pattern to *LAZY1*^19^, which modulated plant branch growth angle, but had opposite functions^19,20^. Several studies have shown that light and gravity signals function concurrently to shape plant architecture^21–23^. A report showed that the expression of *AtTAC1*, which modulated plant architecture in response to photosynthetic signals, was light dependent^24^. The expression of *AtIGT* genes was also found to respond differentially to alterations in light signaling, and the loss of several *LAZY* and *DRO* genes in *Arabidopsis* resulted in a lack of branch angle reaction to light stimuli^25^. These studies have demonstrated that IGT family genes play a role in regulating plant architecture in response to both light and gravity stimuli.

The peanut* (Arachis hypogaea L*.) is one of the world’s most important economic oilseed crops. The peanut seeds are rich in vegetable oils, proteins, vitamins and minerals. Peanuts produce aerial flowers but subterranean fruits (pods). Embryo development remains arrested until the fertilized ovary is buried in the soil with the help of a specialized organ called the peg or gynophore. Positive gravitropism is the most typical features of pegs as it enables them to grow downwards and penetrate in the soil^26^. Then the height of flowers directly influences pegs to penetrate the soil and affect peanut yield. As a result, optimizing plant architecture improves planting density and increases pod number per plant in peanut. Although the critical importance of *IGT* genes involved in regulating gravitropism and shaping the structure of plants^27–29^, little is known about this gene family in *A. hypogaea*. This study is the first genome-wide study to identify the *IGT* genes in the genome of peanuts. To gain a better understanding of *IGT* genes in peanuts, their phylogenetic relationships, chromosome locations, synteny study, gene structures, conserved motifs, and *cis*-element were examined. Additionally, their expression profiles in numerous tissues and their response to light and dark signals were evaluated. The results may facilitate functional studies of the *IGT* genes in peanut.

## Materials and methods

### Identification and characterization of IGT genes in peanut

The protein and genomic sequences of *A. hypogaea* var. Shitoqi were downloaded from the Peanut Genome Resource (PGR) database (http://peanutgr.fafu.edu.cn/Download.php)^30^. The sequences of seven AtIGTs (protein sequences are listed in supplementary Table S1) were downloaded from the *Arabidopsis* Information Resource (TAIR) database (https://www.arabidopsis.org/). These sequences were then subjected to the blastp program to search for the peanut protein sequence database with an E-value ≤ 1.0e−6. Further, 7 AtIGT protein sequences were subjected to MAFFT version 7.0 software^31^ for multiple sequence alignment. The alignment results were used to build HMM profile using hmmbuild program^32^. Then, the HMM profile was used to screen protein sequences of peanuts on HMMER software^32^ with an E-value ≤ 1.0e−6. The amino acid sequences of candidate AhIGTs from blastp and HMMER search were aligned with those of the corresponding AtIGTs using DNAMAN version 5.0 software (Lynnon Biosoft, San Ramon, CA, USA) to confirm whether they were the orthologs of the target AtIGTs or not. The protein sequences of *Arachis duranensis* and *Arachis ipaensis* were obtained from the PeanutBase database (https://data.legumeinfo.org/Arachis/)^33^, and the IGT proteins in these two species were identified using the blastp method. The sequences of the confirmed proteins were submitted to the ExPASy tool^34^ to predict protein molecular weight (MW) and isoelectric point (PI). Subcellular localization of the proteins was predicted by WoLF PSORT^35^.

The structure of *AhIGT* genes was analyzed by the Gene Structure Display Server 2.0^36^, and conserve protein motifs were predicted by the MEME software^37^ with a maximum number of motifs (10) and motif length ranging from 6 to 20 amino acids. The conserved motifs were visualized using the TBtools software^38^. The final figures prepared for gene structures and conserved motifs were refined using Adobe Illustrator software (Adobe Systems Incorporated, San Jose, CA, USA).

### Chromosome location, phylogenetic tree, and synteny analysis of *AhIGT* genes

The chromosomal location of *AhIGTs* was obtained from the PGR database^30^. The distribution of *AhIGTs* was visualized using MapChart software^39^, and the final figure was refined with Adobe Illustrator software (Adobe Systems Incorporated, San Jose, CA, USA).

The full-length amino acid sequences of IGT proteins from *A. thaliana*, *A. hypogaea*, *A. duranensis* and *A. ipaensis* were submitted to ClustalW software^40^ to perform sequence alignment. Sequence alignment results were used as the basis to construct a phylogenetic tree by maximum likelihood method using MEGA 7.0 software^41^. The phylogenetic tree was visualized by the Evolview website^42^.

The synteny analysis of AhIGTs in *A. hypogaea* was performed with McScanX software and the results were plotted by Circos software^43^. The collinear relationships among IGT proteins in *A. hypogaea*, *A. duranensis* and *A. ipaensis* were determined using the Python version of McScanX^44^. The Ka/Ks values of IGT proteins were calculated using the KaKs calculator 2.0 program^45^.

### Analysis of* cis*-regulatory element distribution in AhIGT gene promoters and GO annotation

The 1500-bp sequences occurring upstream of the start codon of *AhIGTs* were extracted from the PGR database^30^. The putative *cis*-elements of each promoter were analyzed using the PlantCARE database^46^, and the results were visualized using the Gene Structure Display Server 2.0 online tools^36^. The full-length amino acid sequences of AhIGTs were submited to the eggNOG website^47^. GO enrichment analysis was performed using the TBtools software^38^.

### Expression profiles and qRT-PCR analysis of AhIGT genes

The tissue-specific expression profiles were obtained from the PGR database (http://peanutgr.fafu.edu.cn/Transcriptome.php)^30^. The fragment per kilobase per million reads (FPKM) value of 9 different tissues including cotyledon, root, stem, leaves, stem tip, gynophore, testa, pericarp, and embryo were downloaded, and the heatmap was drawn using Heatmapper^48^.

A peanut variety PX05 provided by the Crops Research Institute, Anhui Academy of Agricultural Science, Hefei, China, was used as the plant material and exposed to continuous light and dark conditions. The use of plant parts and seeds in the present study complies with relevant institutional, national, and international guidelines and legislation. The seedlings were grown in a growth chamber at 25–30 ℃ under 14:10 h light/dark conditions. The 5- or 6-week-old seedlings were transferred to chambers in continuous darkness for 48 h, and thereafter, leaves were sampled at 0, 0.5, 1, 2, 4, 8, 12, 24 and 48 h after the treatment. The plants were then transferred to chambers under continuous light, and leaves were sampled at 0.5, 1, 2, 4, 8, 12, 24 and 48 h after the treatment. We combined equal amounts of leaves from 5 plants as a biological replicate, and three biological replicates were considered in our experiment. Total RNA was extracted using the SDS-LiCl method which was described by Vennapusa^49^. We used the PrimeScript™ RT reagent Kit (TaKaRa, Japan) to synthesize cDNA according to developer instructions. qRT-PCR assays were performed using SYBR Premix Ex Taq™ (TaKaRa, Japan) with three technical replicates on a CFX Connect Real-Time PCR Detection System (Bio-Rad, USA). The conditions for the qRT-PCR reaction were set as follows: initial denaturation at 95 ℃ for 5 min, then 40 cycles of denaturation at 95 ℃ for 30 s, annealing at 60 ℃ for 1 min. The quantified data were calculated using the 2^−ΔΔCt^ method. The *Actin* gene from peanut (*A. hypogaea*)^50^ was used as an internal control and all *AhIGT* primers are listed in Supplementary Table S2.

## Results

### Identification and characterization of IGT family genes in peanut

**Figure 1:**
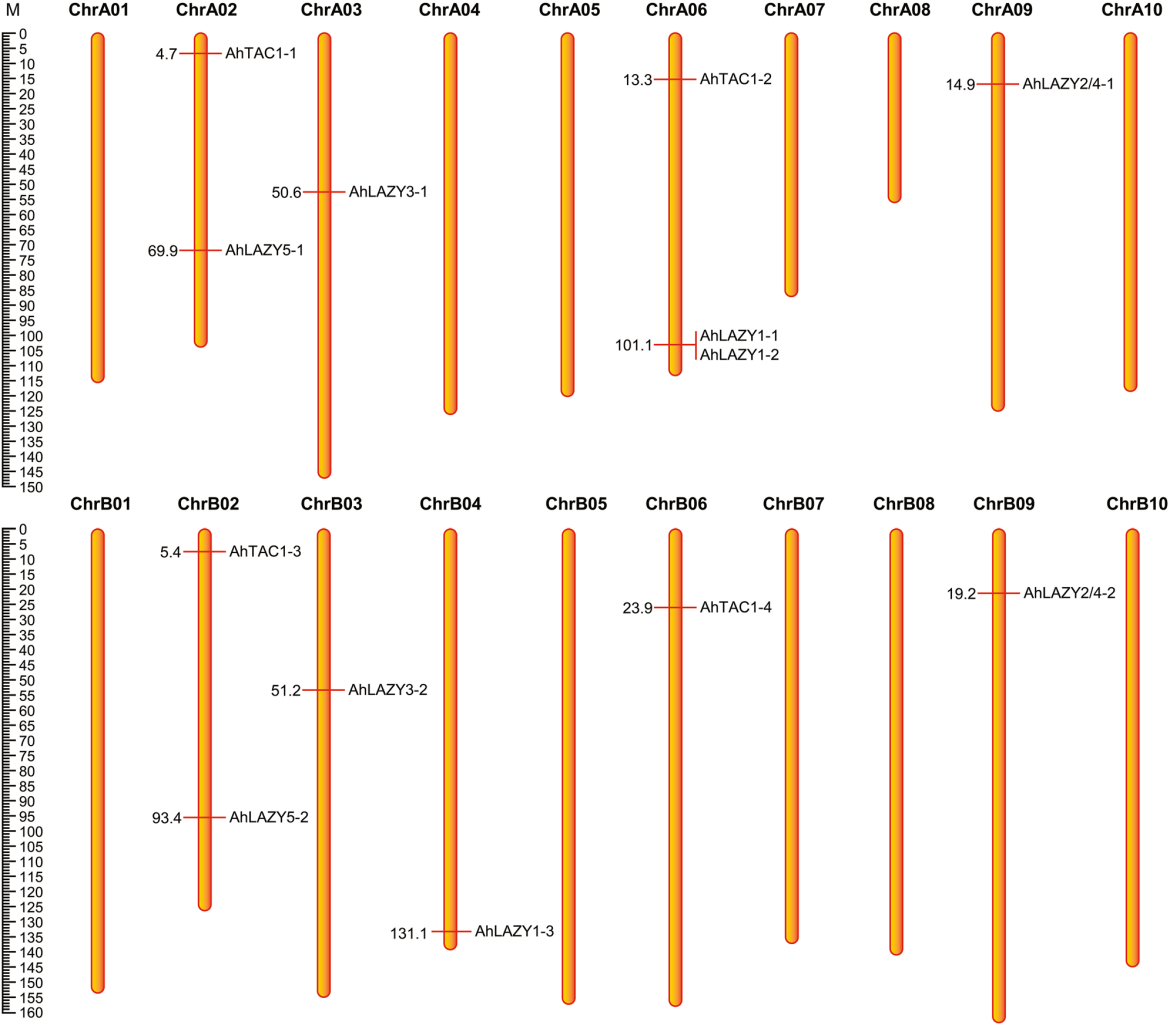
The physical map of *AhIGT* genes located on chromosomes; The diagram was drawn by the Mapchart software, and the physical location of *AhIGTs* was obtained from the Peanut Genome Resource (PGR) database (http://peanutgr.fafu.edu.cn/Download.php).

A total of 13 *IGT* genes unevenly distributed along 9 out of the 20 *A. hypogaea* chromosomes, were identified in peanut genome, with 7 genes located on AA subgenome and 6 located on BB subgenome. Chromosome A06 contained the maximum number of *IGT* genes (3); while chromosomes A02 and B02 each contained 2 *IGT* genes, and chromosomes A03, A09, B03, B04, B06, and B09 each contained 1 *IGT* gene (Table 1, Fig. 1). Most homologous *IGT* gene pairs that had strong collinear relationships were observed to be located on the homologous chromosomes between the AA and BB subgenomes. For example, the *IGT* genes *AhTAC1-1* and *AhLAZY5-1* present on chromosome A02 were found to have strong collinear relationships with the *IGT* genes *AhTAC1-2* and *AhLAZY5-2* present on chromosome B02. The lengths of AhIGT proteins varied from 241 (AhTAC1-2) to 417 (AhLAZY1-2) amino acids, whereas their predicted molecular weight ranged from 27.67 (AhTAC1-2) to 46.50 kDa (AhLAZY1-2), and the isoelectric points ranged from 5.03 (AhLAZY3-1) to 9.73 (AhLAZY5-2) (Table 1). After conducting in silico prediction of subcellular localization, AhTAC1-1 and AhTAC1-3 were shown to be located in the cytoplasm, while the others were located in the nuclear (Table 1). Additional information such as gene position and coding sequence lengths can be found in Table 1.

**Table 1:** Information on the identified *IGT* genes in *A. hypogaea*.

Locus name	Gene name	Genomic position (bp)	CDS (bp)	Protein			Subcellular localization prediction
				Length (aa)	MW (kDa)	PI	
AH02G03950	AhTAC1-1	A02: 4681501–4685322: +	933	310	35.24	5.28	Cytoplasm
AH06G09900	AhTAC1-2	A06: 13253103–13254848: +	726	241	27.67	5.44	Nuclear
AH12G04310	AhTAC1-3	B02: 5359403–5363135: +	939	312	35.36	5.24	Cytoplasm
AH16G14050	AhTAC1-4	B06: 23905432-23907095: +	735	244	27.91	5.1	Nuclear
AH09G10100	AhLAZY2/4-1	A09: 14857638-14860825: −	795	264	30.01	8.43	Nuclear
AH19G13150	AhLAZY2/4-2	B09: 19153113–19156231: −	795	264	30.04	8.24	Nuclear
AH02G17790	AhLAZY5-1	A02: 69884299–69887590: −	858	285	32.47	9.59	Nuclear
AH12G21010	AhLAZY5-2	B02: 93358193–93361544: −	876	291	33.23	9.73	Nuclear
AH06G24530	AhLAZY1-1	A06: 101062830–101065320: +	876	291	32.41	5.39	Nuclear
AH06G24550	AhLAZY1-2	A06: 101070089–101073018: +	1254	417	46.5	6.13	Nuclear
AH14G41310	AhLAZY1-3	B04: 131087855–131092667: −	1176	391	43.62	6.36	Nuclear
AH03G24520	AhLAZY3-1	A03: 50565464–50568947: +	795	264	30.31	5.03	Nuclear
AH13G27590	AhLAZY3-2	B03: 51222944–51226377: +	780	259	29.62	5.21	Nuclear

*CDS* coding sequence, *MW* molecular weight, *PI* isoelectric point.

### Phylogenetic relationships among IGT genes

**Figure 2:**
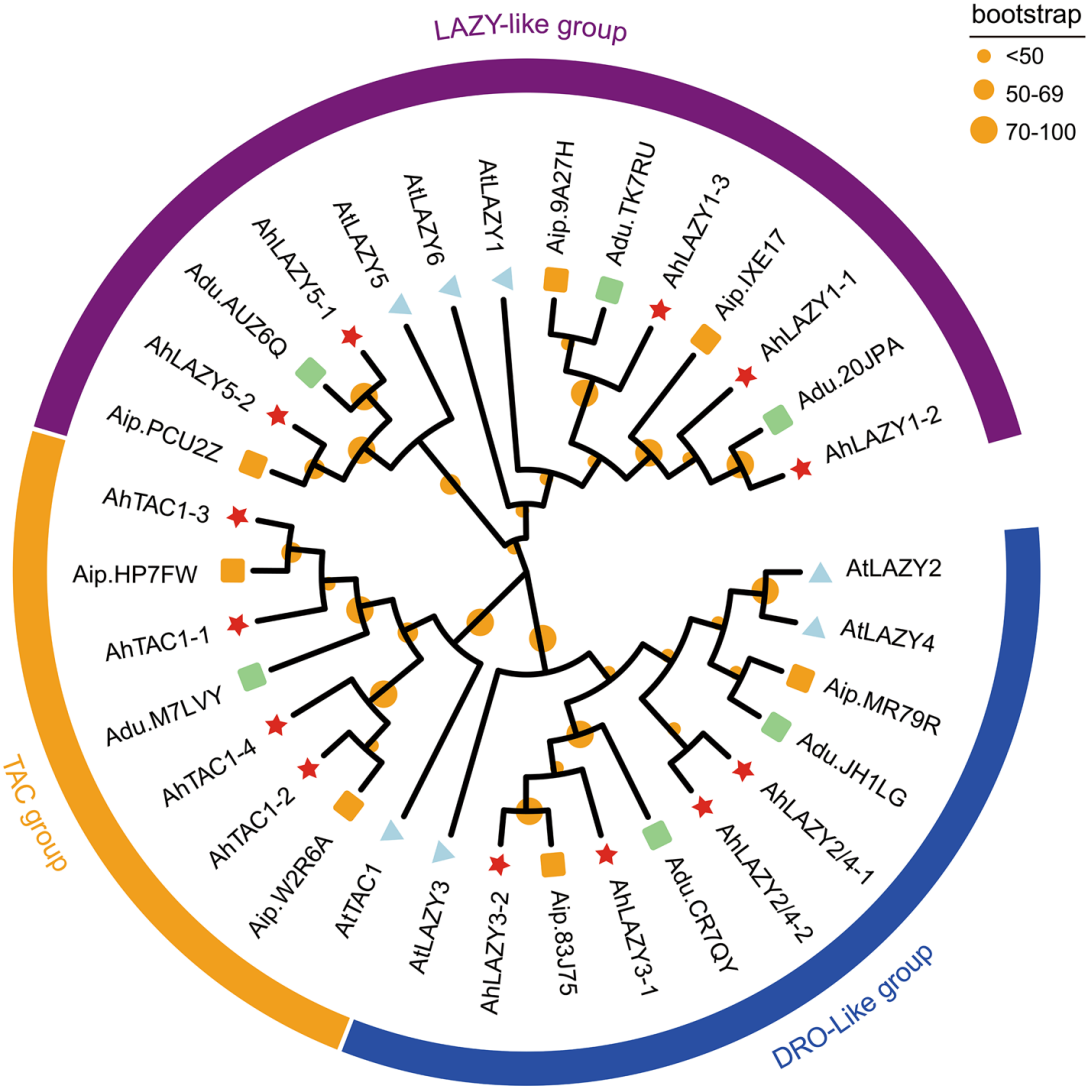
A phylogenetic tree of IGT family genes in *A. thaliana*, *A. hypogaea*, *A. duranensis*, and *A. ipaensis* constructed using the maximum likelihood method; Overall, 13 IGTs from *A. hypogaea* (red stars), 7 IGTs from *A. thaliana* (light blue triangles), 7 IGTs from *A. ipaensis* (orange boxes), and 6 IGTs from *A. duranensis* (light green boxes) were clustered into three major classes, denoted by different colors. The bootstrap is displayed in orange circles.

To investigate the evolutionary relationships among *IGT* genes, a phylogenetic tree was constructed using the sequences of 33 IGT proteins from *A*. *thaliana*, *A. hypogaea*, *A. duranensis*, and *A. ipaensis* (protein sequences are shown in Table S1). As shown in Fig. 2, the tree was divided into three groups (LAZY-like, DRO-like, and TAC). The LAZY-like group included LAZY1, LAZY5, and LAZY6, while the DRO-like group consisted of LAZY2, LAZY3, and LAZY4, and the last group (TAC) only contained TAC1. Most AhIGTs within each group were paired with their orthologs in either *A. ipaensis* or *A. duranensis*. For example, in the TAC1 clade, AhTAC1-1 (A02) was clustered with its *A. duranensis* counterpart Adu.M7LVY (A02), while AhTAC1-3 (B02) was clustered with its ortholog Aip.HP7FW (B02) in *A. ipaensis*. Some AhIGTs like AhLAZY1-3 (B04) had corresponding orthologs both in *A. ipaensis* (Adu.TK7RU, A04) and *A. duranensis*, (Api.9A27H, B04). However, in the DRO-like subgroup, 3 pairs of genes, including AtLAZY2 and AtLAZY4, AhLAZY2/4-1 and AhLAZY2/4-2, and Aip.MR79R and Adu.JH1LG were clustered together. It was difficult to determine which proteins were orthologs of AtLAZY2 or AtLAZY4. AtLAZY6 formed a separate branch, with no ortholog found in *A. hypogaea*, *A. duranensis*, or *A. ipaensis*.

### Gene structures and conserved motifs of IGT genes in peanut

**Figure 3:**
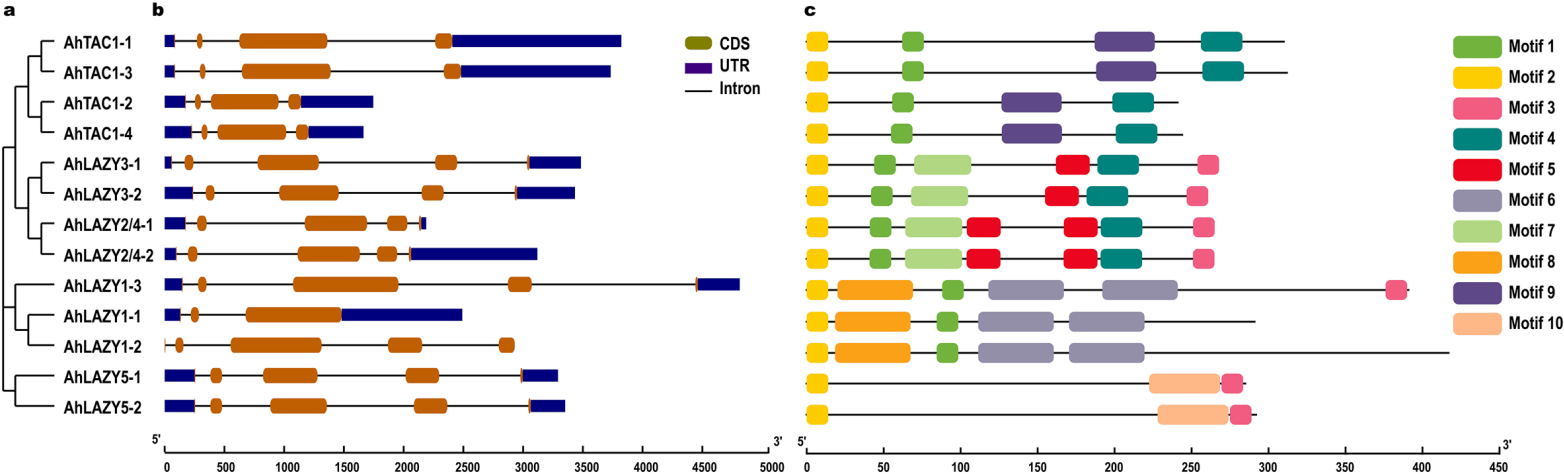
The structures and conserved motifs of *IGT* genes in peanut; (**a**) A phylogenetic tree of *IGT* genes in peanuts was constructed by the MEGA7.0 software. (**b**) The structure of *AhIGT* genes; Blue boxes represent the 5’ or 3’ untranslated regions, while red boxes denote exons and black lines symbolize introns. (**c**) Conserved motifs of AhIGTs; Different colored boxes indicate different motifs.

To have a better understanding of the structure of *AhIGT* genes, the exon–intron arrangements were generated (Fig. 3). The results revealed that most *AhIGT* genes shared similar structural patterns. For example, all 13 *AhIGT* genes consisted of two short exons followed by a long exon. In addition, a short exon was observed to appear at the end of *AhLAZY1-3* and all members of the *AhLAZY2/4*, *AhLAZY3*, *AhLAZY5* clades. Expect for *LAZY1-1*, which had 3 exons, all LAZY-like genes and *DRO-like* genes contained 5 exons, while all *AhTAC1* clade members contained 4 exons.

Furthermore, the full-length amino acid sequences of all AhIGTs were examined to identify their conserved motifs (Fig. 3). As shown in Fig. 3, motif 1 contained the family-specific GφL (A/T) IGT sequence, which was identified in all AhIGT proteins except for the proteins of the AhLAZY5 clade. All AhIGTs contained motif 2 at the N terminus. Motif 3, which contained the EAR motif (Figure S1), was found in all members of AhLAZY3, AhLAZY2/4, and AhLAZY5 clades. However, in AhLAZY1 clade, only AhLAZY1-3 contained motif 3 with the EAR motif. Motif 4 was identified in the TAC1, LAZY2/4, and LAZY3 clades. Motifs 5 and 7 were specific to DRO-like proteins, while motifs 6 and 8 were only specific to the LAZY1 clade. Motif 9 was only detected in the TAC1 clade, whereas motif 10 was only detected in the LAZY5 clade.

### Duplication and synteny analysis of IGT genes in peanut

**Figure 4:**
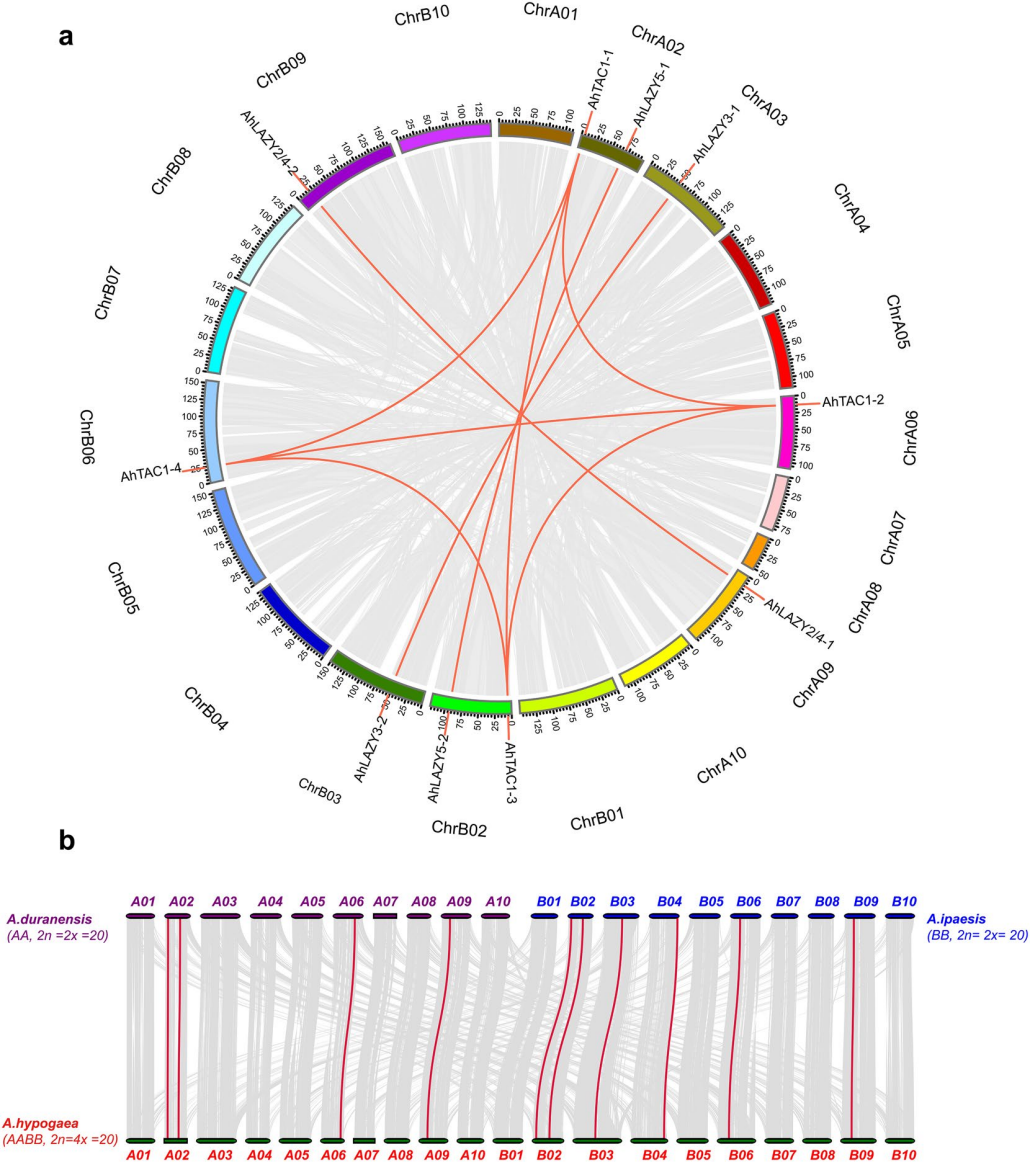
Duplications and synteny analysis of *IGT* genes in peanut; (**a**) Collinearity analysis of AhIGTs in *A. hypogaea*; Grey lines in the background represent all collinear blocks among different chromosomes, while red lines indicate segmentally duplicated gene pairs. (**b**) Collinearity analysis of AhIGTs in *A. hypogaea*, *A. ipaensis* and *A. duranensis*; All collinear blocks among *A. hypogaea*, *A. ipaensis* and *A. duranensis* are represented by grey lines in the background and syntenic *IGT* gene pairs are represented by red lines.

Synteny analysis was performed to explore the evolutionary relationships among *IGT* genes in *A. hypogaea* (Fig. 4A). A total of 9 segmentally duplicated gene pairs were detected, and no tandem duplication was found. After calculating the Ka/Ks ratio, all duplicated IGT gene pairs in peanut were shown to have a Ka/Ks ratio less than 1 (Table 2), indicating that purifying selective pressure acted on evolutionary process of the *AhIGT* genes.

**Table 2:** The Ka and Ks values of duplicated *AhIGT* gene pairs in peanut.

Gene_Name	Gene_Name	Ka	Ks	Ka_Ks	Selection pressure	Duplication type
AhTAC1-1	AhTAC1-2	0.27	1.00	0.27	Purifying selection	Segmental
AhTAC1-1	AhTAC1-3	0.02	0.04	0.47	Purifying selection	Segmental
AhTAC1-1	AhTAC1-4	0.28	0.99	0.29	Purifying selection	Segmental
AhTAC1-2	AhTAC1-3	0.28	1.04	0.27	Purifying selection	Segmental
AhTAC1-2	AhTAC1-4	0.01	0.05	0.22	Purifying selection	Segmental
AhTAC1-3	AhTAC1-4	0.29	1.02	0.29	Purifying selection	Segmental
AhLAZY2/4-1	AhLAZY2/4-2	0.01	0.06	0.14	Purifying selection	Segmental
AhLAZY5-1	AhLAZY5-2	0.01	0.03	0.41	Purifying selection	Segmental
AhLAZY3-1	AhLAZY3-2	0.01	0.03	0.31	Purifying selection	Segmental

Further, the collinearity analysis of genes among *A. hypogaea*, *A. ipaensis* and *A. duranensis* was performed (Fig. 4B). A total of 10 AhIGTs in *A. ipaensis* and *A. duranensis* showed the correspondent relationships. Four AhIGTs in the AA subgenome exhibited a one-to-one homologous relationship with their orthologs in *A. duranensis*, and 6 AhIGTs in the BB subgenome had a one-to-one homology relationship with their orthologs in *A. ipaensis*. No IGT gene pair was found between the AA subgenome and the *A. ipaensis* genome or between the BB subgenome and the *A. ipaensis* genome. The results indicated that gene duplication played a crucial role in the evolution of the IGT gene family in the peanut genome. The Ka/Ks ratio was also determined for *A. hypogaea* and its two diploid ancestors (Table S3). All duplicated gene pairs had a Ka/Ks ratio < 1, indicating the role of strong purifying selective pressure in the evolutionary process of *IGT* genes in these species.

### *Cis*-element analysis and functional annotation of *IGT* genes in peanut

**Figure 5:**
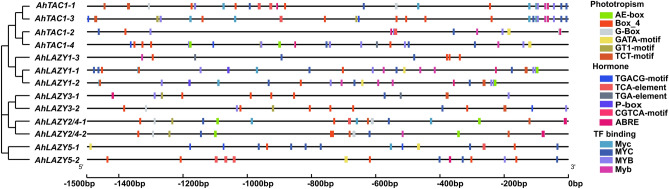
*Cis*-element analysis of IGT genes in *A. hypogaea*. Different color boxes represent different *cis*-elements.

The 1500-bp upstream sequences of *AhIGT* genes were extracted to perform the *cis*-element analysis. Apart from the general transcriptional regulators and elements with unknown functions, most *cis*-elements could group into three categories, which involved light-responsive elements, hormone-responsive elements, and transcription factor (TF)-binding elements (Fig. 5). Light-responsive elements included AE-box, Box4, G-box, GA-motif, GATA-motif, GT1-motif, TCT-motif, chs-CMA1a, chs-CMA2a and Sp1. Hormone-responsive elements included CGTCA-motif and TGACG-motif (Methyl jasmonate, MeJA), P-box (gibberellic acid, GA), TCA-element (salicylic acid, SA), ABRE (abscisic acid, ABA) and TGA-element (auxin). TF-binding elements, however, included Myb, MYB, Myc, MYC. Interestingly, the light-responsive elements accounted for the majority of all *cis*-acting elements, which indicated that *AhIGT* genes may play roles in the regulation of photoactivity.

GO annotation and GO enrichment analysis of AhIGTs were performed to gain an understanding of their functions (Table S4). Only three members of AhLAZY1 were annotated, which were found to be involved in the biological process (BP) and the cellular component (CC). In the biological process, AhLAZY1-3 was annotated in gene ontology (GO) terms of hormone transport and regulation of polar auxin transport, whereas AhLAZY1-1 and AhLAZY1-2 were annotated in gravitropism. In the cellular component, three annotated genes were located in the nucleus, which was consistent with the subcellular location prediction of these genes.

### Expression patterns of AhIGT genes in various tissues

**Figure 6:**
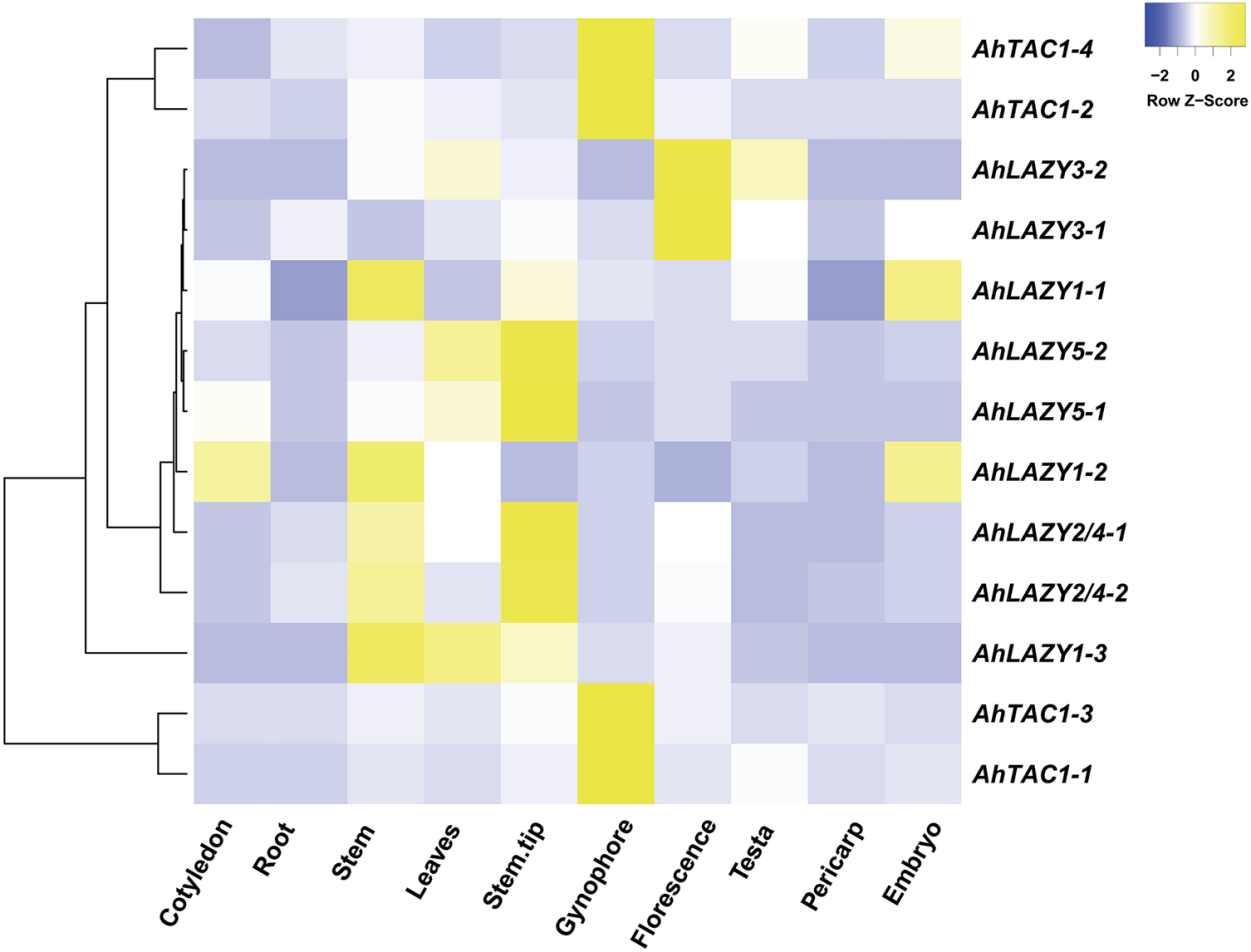
Expression profiles of *AhIGT* genes in various tissues; The transcriptome data were obtained from the PGR database. The heat map was created using the log2 based FPKM expression values.

To explore the expression patterns of *AhIGT* genes in different tissues, we downloaded the transcriptome profiles of 13 *AhIGT* genes across 9 different tissues from the Peanut Genome Resource (PGR) database. As shown in Fig. 6 and Table S5, some *AhIGT* genes displayed relatively lower expression. For example, *AhLAZY3-1*, *AhLAZY3-2*, *AhLAZY5-1*, *AhLAZY5-2*, and *AhAZY1-1* had FPKM values less than 1 in all 9 tissues. In addition, most *AhIGT* genes were also observed to have relatively lower FPKM values in cotyledon, root, testa, pericarp, and embryo. High expression of *AhIGTs* was usually detected in the stem, leaves, and stem tip. For example, *AhLAZY1-3* had the highest FPKM values in the stem, while *AhLAZY2/4-1* and *AhLAZY2/4-2* had the highest FPKM values in the stem tip. These genes also exhibited relatively higher expression in leaves or the stem. The highest expression of *AhTAC1s* was detected in the gynophore, and their relatively higher expression was observed in the stem, leaves and some other tissues.

### Expression patterns of *AhIGT* genes in continuous darkness and light

**Figure 7:**
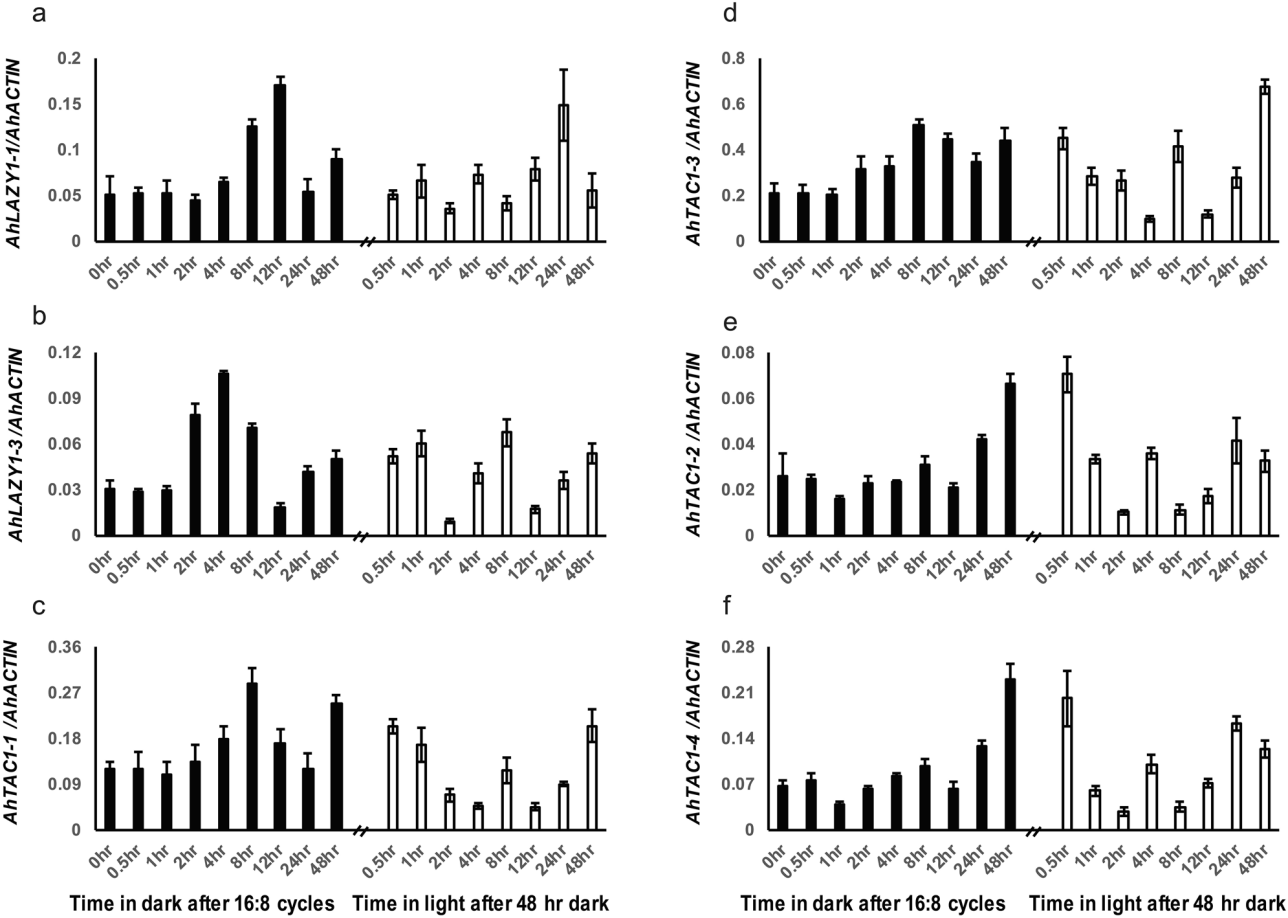
Expression patterns of *AhIGTs* under continuous darkness and light; Dark and light conditions represented by black and white columns, respectively. Error bars represent the standard deviation (SD).

The* cis*-elements analysis showed that the light-responsive elements accounted for the majority of all *cis*-acting elements in the promoter region of *AhIGT* genes. Next, we explored the dynamic expression profiles of these genes under continuous dark and light conditions (Fig. 7). A total of 9 genes including *AhLAZY1-1*, *AhLAZY1-3*, *AhLAZY3-1*, four paralogs of *AhTAC1* and two paralogs of *AhLAZY5* were evaluated, out of which three (*AhLAZY3-1* and two paralogs of *AhLAZY5*) exhibited no expression in leaves.

In continuous darkness, the expression of *AhLAZY1-1* increased after 4 h of the treatment and peaked at 12 h, followed by a decrease thereafter and an increase after 24 h of darkness (Fig. 7A). Similarly, the expression of *AhLAZY1-3* increased after 1 h, peaked at 4 h, and then declined, with a final increase after 12 h of dark treatment (Fig. 7B). The expressions of both *AhTAC1-1* and *AhTAC1-3* were variable (Fig. 7C, D). However, *AhTAC1-2* and *AhTAC1-4* showed different expression patterns. Although their expression showed a relatively lower level at 1 and 12 h, it started increasing in continuous darkness and peaked at 48 h (Fig. 7E, F).

In continuous light, the expressions of both *AhLAZY1-1* and *AhLAZY1-3* were variable (Fig. 7A, B). *AhTAC1-1* and *AhTAC1-3* showed similar expression patterns, i.e., their expression was first downregulated gradually and then upregulated in a stepwise manner in the late stage. In addition, both these two genes demonstrated sharply increased expression at 8 h (Fig. 7C, D). Similar expression patterns with little difference were also observed in *AhTAC1-2* and *AhTAC1-4*, with a sharp decrease after 30 min, followed by a gradual decline after 24 h (Fig. 7E, F).

## Discussion

The IGT family genes are known as key players in gravity sensing, which is critical to the optimization of crop architecture^1^. Currently, 4, 2, 27 and 4 members of the IGT gene family have been identified in rice^27^, maize^1^, rapeseeds^28^ and apple^29^. In this study, 13 *AhIGT* genes were identified, unevenly distributed on 9 chromosomes. The 13 *AhIGT* genes could be classified into three group, consistent with previous studies’ results^1,25^. WGD is recognized as a major driving force of plant evolution, resulting in gene duplication and subsequent silencing and removal of duplicate genes^51^. Phylogenetic, chromosomal locations and synteny analysis revealed strong collinear relationships among most *IGT* genes in *A*. *hypogaea* between the AA and BB subgenome. Additionally, most *AhIGT* genes in the AA and BB subgenomes showed corresponding relationship with those in *A*. *duranensis* and *A*. *ipaensis*, respectively. These indicated the crucial role of segmental duplications in the expansion of the IGT gene family. Although tetraploid peanut contains the same number of *IGT* genes in *A*. *duranensis* and *A*. *ipaensis* combined, the number of *IGT* homologous genes between their subgenome in *A*. *hypogaea* and its two wild progenitors was not equivalent. This suggests that the chromosome doubling and then sequence arrangement would cause the loss of *IGT* genes during the polyploidization process.

In *Arabidopsis*, 5 short conserved regions and a unique intro-exon arrangement were presented in *IGT* family genes^1,52^. These conserved regions and intro-exon arrangement are also identified in *IGT* family genes in rice^27^, rapeseeds^28^ and apple^29^. In this study, motif 1, 2 and 3 corresponded exactly to the conserved regions II, I and V, and a similar intro-exon arrangement was also presented in *AhIGT* family. The exon–intron arrangement could also explain the evolutionary history of the gene family^53^. It is wide accepted that homologous proteins with similar gene structures and conserved motifs across different species may possess identical or similar functions^1,52,54^. So, it is conjectured that AhIGTs might participated gravity sensing and shaping plant architecture in peanuts.

Using public databases^30^, the expression patterns of *AhIGTs* across 9 different tissues were obtained. Interestingly, the expression level of *AhLAZY1-3* in the stem tip and stem was much greater than that of the other two paralogs *AhLAZY1-1* and *AhLAZY1-2*. GO annotation analysis indicated that *AhLAZY1-3*, not *AhLAZY1-1/2*, were annotated in the regulation of auxin polar transport. It is known that *IGT* genes regulate the gravitropic responses through asymmetrical auxin distribution^1,15,52^. In the meantime, only *AhLAZY1-3* contained motif 3 which is a key domain of *AtLAZY1* in *Arabidopsis*^6^. Hence, it is reasonable to conjecture that *AhLAZY1-3* might participate in gravity sensing and shaping peanut architecture. However, this prediction needs to be tested by more experimental evidence. A much higher expression level of all *AhTAC1* genes was found in the gynophore. Gynophore in peanuts is specialized to develop peg which is essential for burying the fertilized ovary into the soil. Positive gravitropism is the most typical features of pegs^26^. Whether *AhTAC1s* participate in peg penetration into the soil is another interesting project supported by further experimental evidence in the future.

*Cis*-element analysis showed that light-responsive elements accounted for most *cis*-acting elements in the promoter of *AhIGTs*. Several recent studies have reported that the signals of light and gravity function together in shaping plant architecture^21–23^. In *Arabidopsis*, the expression of *TAC1* gradually declined once plants were transferred into the dark and returned to their normal levels 48 h after it was transferred back into the light^24^. The expression of *TAC1*, which plays an important role in regulating plant architecture in response to light signals, was light dependent^24^. qRT-PCR revealed that the expression of *AhTAC1-2/4* was also light dependent but with a different pattern. *AhTAC1-2/4* expression increased in the dark and then decreased sharply once transferred back in the light. Hence, it is speculated that *AhTAC1–2/4* may have regulated plant architecture in response to light signals.

## Conclusions

In this study, a genome-wide analysis of IGT family genes was performed in the genome of cultivated. A total of 13 *AhIGT* genes, which were found to be unevenly distributed on 9 chromosomes and classified into three groups, were identified. Gene structure, conserved motifs, and *cis*-element were examined to gain insights into *IGT* genes in peanuts. Their expression profiles across different tissues, and under different light and dark conditions were also evaluated. Results revealed that *AhLAZY1-3* may play roles in shaping peanut architecture, *AhTAC1* could potentially be involved in the process of peg penetration into the soil, and several *AhIGT* genes, like *AhTAC1-2/4* may regulate plant architecture in response to gravity signals. The results provide a theoretical basis for further characterization of the biological functions of the *AhIGT* genes in shaping plant architecture.

### Supplementary Information

Supplementary Information.

## Data Availability

The data used in this article are open and publicly available. The links to the databases are listed below. Peanut Genome Resource (http://peanutgr.fafu.edu.cn/Download.php). The *Arabidopsis* Information Resource (TAIR) database (https://www.arabidopsis.org/). The PeanutBase (https://peanutbase.org). WoLF PSORT (https://www.genscript.com/wolf-psort.html). PlantCARE website (http://bioinformatics.psb.ugent.be/webtools/plantcare/html). ProtParam tool of ExPaSy (https://web.expasy.org/protparam/).
