# Supplementary material for: Genome-wide identification of peanut IGT family genes and their potential roles in the development of plant architecture
Source: Sci Rep. 2023 Nov 21;13:20400. doi: 10.1038/s41598-023-47722-4 (PMC10663514; doi:10.1038/s41598-023-47722-4)
Supplement: Supplementary file 1 — Supplementary Information. [file 41598_2023_47722_MOESM1_ESM.docx]

**Supporting Information**

Genome-wide identification of peanut IGT family genes and their potential roles in plant architecture development

**Wen Chu ^1^, Xiaofeng Zhu ^1^, Tao Jiang^1^, Song Wang^1^ and** **Wanli Ni ^1,^***

1 Crops Research Institute, Anhui Academy of Agricultural Sciences, Hefei, 230031, China

* Correspondence:

Wanli Ni

[wlpeanut@163.com](mailto:wlpeanut@163.com)

Table S1: The amino acid sequence of IGT proteins from *A*. *thaliana*, *A. hypogaea*, *A. duranenesis*, and *A. ipaensis*

| **Gene name** | **Sequences** |
| --- | --- |
| AtTAC1  (At2g46640) | MTIFNWVQKKLHQNVIKEIDGVRKNEKKKRNEGISEIEKNTKAILDQVGLVDALDNWFDGVLTIGTFGFDTLNFKEEDEMDDDECGSGDLDYVVIDGSIIKNVDQESDPLISVENKFYDHHEDVGNLYVQADTDHFGSIKTAETPAVTAAAEAEIEPQKKRTTLAELFMEDHDKGYDTWHCKKPNNPNLDGEEVKYYKQNGSKLSKRFSFVKKKLVMSKSKEEENDLRPIKKMRQMIKRMLKKKIHPDVDATKALKKDVPYKPTRNCEALESHYLLKIQDCVA |
| AtLAZY2  (AT1G17400) | MKFFGWMQNKLNGDHNRTSTSSASSHHVKQEPREEFSDWPHALLAIGTFGTTSNSVSENESKNVHEEIEAEKKCTAQSEQEEEPSSSVNLEDFTPEEVGKLQKELMKLLSRTKKRKSDVNRELMKNLPLDRFLNCPSSLEVDRRISNALSAVVDSSEENKEEDMERTINVILGRCKEISIESKNNKKKRDISKNSVSYLFKKIFVCADGISTAPSPSLRDTLQESRMEKLLKMMLHKKINAQASSKPTSLTTKRYLQDKKQLSLKSEEEETSERRSSSDGYKWVKTDSDFIVLEI |
| AtLAZY3  (AT1G19115) | MKIFSWVQRKLSGKKRVPTSDSSQEPSSPPLSKEVQGLPQDEETFLAIGTLGNNIFPKQEEEEEETDSSKDITPVNTDVTIGKKKSLSFLLKKMFVCTSGFKTPPPLLDLSRGDSLHNTRMEKMLRTILNKKIHPQRSNSIAKKYLESNHKIMDEARSSVDANKWVKTDSECEIF |
| AtLAZY4  (At1g72490) | MKFFGWMQNKLHGKQEITHRPSISSASSHHPREEFNDWPHGLLAIGTFGNKKQTPQTLDQEVIQEETVSNLHVEGRQAQDTDQELSSSDDLEEDFTPEEVGKLQKELTKLLTRRSKKRKSDVNRELANLPLDRFLNCPSSLEVDRRISNALCDEKEEDIERTISVILGRCKAISTESKNKTKKNKRDLSKTSVSHLLKKMFVCTEGFSPVPRPILRDTFQETRMEKLLRMMLHKKVNTQASSKQTSTKKYLQDKQQLSLKNEEEEGRSSNDGGKWVKTDSDFIVLEI |
| AtLAZY5  (AT3G24750) | MAKIKAQGGEEQVTSTRFDLKKAIRVLQPEKQDDTNKEKSKSEQSKGKATLSRMKELIKWAAAAKSDKAVKFFTPKIMMELKNRRKLKMMREVNEEESTKRMSSVSANISLRWESSESCTTNSSSDHISIVSSPGILVSLSPTPLYRCRSRKCNWITTDSEFVVLELEL |
| AtLAZY6  (AT3G27025) | MKLLSWMRTIKPNGLDSSKKFKGGLCSLRAQVFSDVQDIRTNSFSFYGHTHDPNPSKVEQDLRFDEDEFCGFLAIGTLGTDPETPKFSAMVAEEDATGEIKEMAKLIAKKLDQFLKEYPEDTRSKRVKISNECPLQDYDLFRSSIELTKGSNGRVKKKKSLLTSLFKRRQTVQGEPYIEKHSTRDAIKRVFKKLHGASSKTRNDDEDDSMSKKKKDLKKNVQTCRRKVHPVLCTTAIPQDDNEIDDRRKVDLKVPSLTGGFLGASSISEANRKRENWIKTDTEYLVLEL |
| AtLAZY1  (At5g14090) | MKFWGWMHHKFRENSKEPLKDASTGNSYSILSAHPSLDSQEVYPTACAGSRYNTGFRKQVNLFQESSFAGPKQYTEEDFKDERNSDFFDGFLAIGTLGGETLLDEQPATPTFGMSFEDPAIDDADVTENDLKLISNELDKFLEAEAKEGHHQPSGRNSDTNTIASTIEAIEGVDDEEDNQPMKFPLQEYFFGSLIELPESKIAGKKDRASLGELFQITEVQDKQSENIYGKKKKQPNSAHKSAKHLVKKVLKKIHPSSRGSVSGKPEVDSTKKKFQKMVQVFHRKVHPEESIMETKIYSS VANPKSSKANSIDLTFEKVNHCHEASKRCIQYELRSSRSAKNGEHWIKTDEDYFVLEL |
| Araip.83J75 | MRHYEPCKQEFGDWPQALLAIGTFGTNSDNQKEDSEKTNKGRALDPPCFQDCEDEFTLEEVGSLQNEFNKYFQVPEEPNSAAKPLSTENSSLEEEGHYGDFHQSSSLMDAKGKDQCLDHSRKNGVAKKSLNFLLKKMLVCNSGFQPTTPFLKDPSLSTESRMEKILRAILNKKIYPQGTSSTMFIKKYLETKSIPQYYSDDDDNDDDEFAKAVVNGCKWVKTDAEYIVLEI |
| Araip.9A27H | MKLLGWMHRKFRQNNSSEPFKDLVIGNSCNCLSGQSPLDDEQNYLKPNNHGVKLFKNMNQKVMRKSFAGIDSTKEHEDYYIEPSSMCDDDLFPGFLAIGTLGSSSDQVVSDPTSTPTFAISVESITEKEDEVTENDLKLINDELEKVLGADDVSIDYYSSGRNSHVSTGRNSHVSTGRSSHVSIITLSGKPITETHNNNNGGSVCPLQGYLFGTAIELSETTRTSSSSSSTMTAAATATATTKKEHRTSLGELFQRSKLAEECAKNNNENKEEKREGDKSAMNLVKEKLKKRILHHASSRNSTSMNDSSSSAAETKLNKILHMFRKKVHPESSTAAHKYSKQHRKNENKKKIMSDIKGSEIVHPDYDENEDSFINREHWIKTDADYLVLEL |
| Araip.HP7FW | MKIFNWVHKRFNHKPPKDGFASDMKKNEVTISNDDNNKVVGDNEVALLKQVALTNMLGGWKDGILTIGTLGYDHKEYFGLQVQDKKLLGVNPLMLNTFEHHNNNFDDDDDDDEEVHVGGGANQIIIDANYNVMTKEEVLINNEIVLADESNDDDDDGESDDVDDDQKKKIVRGERITLADLFLADSEVKKKIMGPTKILVESNDDEKSNLKVKHGKSFAKKLLPNVKDNPQPMKDIKKLMKKMLKRKIHPDFDAKNQKPEKKESIDGNHTKKKEGRKASIYCIPI |
| Araip.IXE17 | MKIFQWVHRKLRQNSIDPFKDFTLANPCSCLTPQPTCDNQYSHMMPSFGSMNQPGFLKPHHQESLTSYSGLDVEGESKQETPAAISELFEGFLTIGTLGIETVNNEPATPTFPMPIEDITMNNAEVTENDLKLISYELEKFLEAEKEESFYESSGRNSQVSTITLSSKQIDGSEAEDNANKAVCPLQGYLLGSSLELPETTEVKKERASLAELFRRTKTSQESIETGEKGETQIKQTQKSAMHIMRKMLKKVHVSSKSCNTAARDDAASTSTNKKLHKVLRMFHRKVYPENTVTAKDFAKPNKDKIKDASHDCCHEYQNRDPTNPDKGRRVNSDTKSTKSSRNCEPIWNPPQDGLSCSTSTANNEHWIKTDAECKYLSL |
| Araip.MR79R | MKFLSWMQNKFSGNQGSTKQNSCTTTTTYHAKQEPREEFSDWPHGLLAIGTFGNNSEIKESQENQNTCSEEPSSSEEIADFTPEEIGKLQKELTKLLRRKPSVEKEISELPLDRFLNCPSSLEVDRRISNALCSDSGDKEEDIEKTLSVILGKCKDICADNSKKAIGKKSLSFLLKKIFVCRSGFAPTPSLRDTLQESRMEKLLRTILHKKICTQNSSRSPVMKKCIQDKKSATKKRNMEEPEERKDDGCKWVKTDSEYIVLEI |
| Araip.PCU2Z | MQILQWVLFKRVHEQKGECNISDTPTKREATSTTYKKGKGKDIVALKHYSRRYKRTKRVEESKIGCIRLKAFAFICGKDIPKACFYRTLNLNKRRDFVHSMKMKREELSRGVGITKNEDSSSSSASQNVGNKKVLPITEGSSTNSSERSEQHFKTVENKKNKKKSISKMKEILRWAAAAKSDKSRRKFNGQKVLEFRRDGTTKSSSVKNEDDEVGIESPKISFSLDMERCSTTYSVYSSATSTDSYSFIENQRAHIAHSNINVSVQGCGYTNNCRHENWITTDSECKPP |
| Araip.W2R6A | MKIVNWVHKRFHHSTTLKDGFASNMKNIEPMRSNNEDSEGMMKQVALAELFGGWKDGILTIGTLGCDPLINSYSQNKQYYALESEEDQEEEEEQEDEENYNGEDDNEEVNPLMQSTFEEVKKVDVTDENSIEEMEKKKKGERITLADLFLADSDVKMEGAKSKVSTADEDEKQSSIMKGKHHMHALSFTKKLIPRGLNKDNPHPIQDIKKLIKKMLKRKIHPELLDVKNPKTSDTNDTASLLLI |
| Aradu.20JPA | LHHHHFSLLQIFQWVHRKLRQNSIDPFKDFTLANPCSCLTPQPTCDNQYSHMMPSFGSMNQPGFLKPHHQESLTSYSGLDVEGESKQETPAAISELFEGFLTIGTLGTEIVNNEPATPTFPMPIEDITMNNAEVTENDLKLISYELEKFLEAEKEESFYESSGRNSQVSTITLSSKQIAGSEAEDNANKAVCPLQGYLLGSSPELPETTEVKKERASLAELFHRTKTSQESIETGEKGETQIKQTQKSAMHIMRKMLKKVHVSSKSCNTAARDDAASASTNKKLHKVLRMFHRKVYPENTVTAKDFAKPNKDKIKDASHDCCHEYQNRDPTNPDKGRRVNSDTKSTKSSPNCEPIWNPPQDGLSCSTSTTNNEHWIKTDAECKYLSL |
| Aradu.AUZ6Q | MQILQWVLFKRVHEQKGECNISDTPTKREATSKGKDIVALKHYRRYKRTKRVEESKIGCIRLKAFAFICGKDIPKACFYRTLNLNKRRDFVHSMKSKREELSRGVGVTKNEDSSSASQNVGNKKVLPITEGSSSNSSERSEQHFKTVENKNNKTKSISKMKEILRWAAAAKSDKSRRKFNGQKVLEFRRDGTIKSSSVKNEDDEVGIESPKISFSLDLERCSTTYSVYSSATSTDSYSFIENQRAHIAHSNINVSGAVIPTIVDMKIGSLLTPNNIISLFCGAGTMKKLNVYKEDILGTPNFTSRASFHQKVVREIN |
| Aradu.CR7QY | MRHYEPCKQEFGDWPQALLAIGTFGTNSDNQKEDSEKTNKGRALDPPCFQDCEDEFTLEEVGSLQNEFNKYFQVPEEPNSAAKPLSTENSSFEEEEEEEEGHYGDFHQSSSLMDAKGKDQCLDHSRKNGVAKKSLNFLLKKMLVCNSGFQPTPPFLKDPSLSTESRMEKILRAILNKKIYPQGTSSTMFIKKYLETKSMPQYYSDDDDNNDDEFAKAVVNGCKWVKTDAECKFSTMI |
| Aradu.JH1LG | MKFLSWMQNKFSGNQGSTKQNSCTTTTTYHAKQEPREEFSDWPHGLLAIGTFGNNSEIKESQEKQNTCSEDPSSSEEIADFTPEEIGKLQKELTKLLRRKPSVEKEISELPLDRFLNCPSSLEVDRRISNALCSDSGDKEEDIEKTLSVILGKCKDICADNSKKAIGKKSLSFLLKKIFVCRSGFAPTPSLRDTLQESRMEKLLRTILHKKICTQNSSRSPVMKKCIQDKKSATKKRNVEEAEERKDDGCKWVKTDSEYLKKSKIQYIKTLTRERKNERKPKGGG |
| Aradu.M7LVY | MKKNEVTISNDDNKVVGDNEVALLKQVALTNMLGGWKDGILTIGTLGYDHKEYFGLQVQDKKLLGGDEGLNKNNNINNNNVFDDYAENDDEEVNPLMLNTFEHHNNNFNDEEEEEEEEEVLVGGGANQIIIDANYNVITKEEVLMNNEIVLADESNDDDDDGESDDVDDDQKKKIVRGERITLADLFLADSEVKKKMMGPTKILVESNDDEKSNLKVKHGKSFAKKLLPNVKDNPQPMKDIKKLMKKMLKRKIHPDFDAKNQKPEKKESIDGNHTKKKEGRKASLYCIPI |
| Aradu.TK7RU | MKLLGWMHRKFRQNNSSEPFKDLVIGNSCNCLSGQSPLDDEQNYLKPNNHGVKLFKNMMMNQKVMRKSFAGIDSTKETEDYYIEPSSMCDDDLFPGFLAIGTLGSSSDQVVSDPTSTPTFAISVESITQKEDEVTENDLKLINDELEKVLGTETNDDVSIDYYSSGRNSHVSTGRNSHVSTGRSSHVSIITLSGKPITETHNNNNGGSVCPLQGYLFGTAIELSETTRTSSSSSSTMTAAATATTTKKEHRTSLGELFQRSKLAEEEGDKSAMNMVKEKLKKRILHHASSRNSTSMNDSSSCAAETKLNKILHMFRKKVHPESSTAAHKYSKQHRKNENKKKIMSDIKGSEMVHPDYDENEDSFINREHWIKTDADCK |
| AH02G03950 | MKIFNWVHKRFNHKPPKDGFASDMKKNEVTISNDDNKVVGDNEVALLKQVALTNMLGGWKDGILTIGTLGYDHKEYFGLQVQDKKLLGGDEGLNKNKNINNNNVFDDYAENDDEEVNPLMLNTFEHHNNNFNDEEEEEEEVLVGGGANQIIIDANYNVITKEEVLMNNEIVLADESNDDDDGESDDVDDDQKKKIVRGERITLADLFLADSEVKKKMMGPTKILVESNDDEKSNLKVKHGKSFAKKLLPNAKDNPQPMKDIKKLMKKMLKRKIHPDFDAKNQKPEKKESIDGNHTKKKEGRKASLYCIPI |
| AH02G17790 | MQILQWVLFKRVHEQKGECNISDTPTKREATSTTCKGKDIVALKHYRRYKRTKRVEESKIGCIRLKAFAFICGKDIPKACFYRTLNLNKRRDFVHSMKSKREELSRGVGVTKNEDSSSASQNVGNKKVLPITEGSSSNSSERSEQHFKTVENKNNKTKSISKMKEILRWAAAAKSDKSRRKFNGQKVLEFRRDGTIKSSSVKNEDDEVGIESPKISFSLDLERCSTTYSVYSSATSTDSYSFIENQRAHIAHSNINVSVQGCGYTNNCRHENWITTDSEFVVLEL |
| AH03G24520 | MKILQWMQKRINGNNEKKKPSPISTTYYMRHYEPCKQEFGDWPQALLAIGTFGTNSDNQKEDSEKTNKGRALDPPCFQDCEDEFTLEEVGSLQNEFNKYFQVPEEPNSAAKPLSTENSSFEEEEEEEEGHYGDFHQSSSLMDAKGKDQCLDHSRKNGVAKKSLNFLLKKMLVCNSGFQPTPPFLKDPSLSTESRMEKILRAILNKKIYPQGTSSTMFIKKYLETKSMPQYYSDDDDNNDDEFAKAVVNGCKWVKTDAEYIVLEI |
| AH06G09900 | MKIFNWVHKRFHHSTNLKDGFASNMKNIEPTRSNNEDSEGMMKQVALAELFGGWKDGILTIGTLGCDPLMNSYSQNKQYYALESEEDQEEEEEQEDEENYNGEDDNEVNPLMQSTLEEVKKVDVTDENSIEEMEKKKKGERITLADLFLADSDVKMEGAKSKDEKQSSIMKGKHHNHAHMHALSFTKKLIPRGLNKDNPHPIQDIKKLMKKMLKRKIHPELLDVKNPKTSDTNDTASLLLI |
| AH06G24530 | MKIFQWVHRKLRQNSIDPFKDFTLANPCSCLTPQPTCDNQYSHMMPSFGSMNQPGFLKPHHQESLTSYSGLDVEGESKQETPAAISELFEGFLTIGTLGTEIVNNEPATPTFPMPIEDITMNNAEVTENDLKLISYELEKFLEAEKEESFYESSGRNSQVSTITLSSKQIAGSEAEDNANKAVCPLQGYLLGSSPELPETTEVKKERASLAELFHRTKTSQESIETGEKGETQIKQTQKSAMHIMRKMLKKVHVSSKSCNTAARDDAASASTNKKLHTSEFICNIKEPKDN |
| AH06G24550 | MKIFQWVHRKLRQNSIDPFKDFTLANPCSCLTPQPTCDNQYSHMMPSFGSMNQPGFLKPHHQESLTSYSGLDVEGESKQETPAAISELFEGFLTIGTLGTEIVNNEPATPTFPMPIEDITMNNAEVTENDLKLISYELEKFLEAEKEESFYESSGRNSQVSTITLSSKQIAGSEAEDNANKAVCPLQGYLLGSSPELPETTEVKKERASLAELFHRTKTSQESIETGEKGETQIKQTQKSAMHIMRKMLKKVHVSSKSCNTAARDDAASASTNKKLHKVLRMFHRKVYPENTVTAKDFAKPNKDKIKDASHDCCHEYQNRDPTNPDKGRRVNSDTKSTKSSPNCEPIWNPPQDGLSCSTSTTNNEHWIKTDAESLVSKWSISDSGAEWLAPKCHHGIANSGIATISRKTENDVYAHN |
| AH09G10100 | MKFLSWMQNKFSGNQGSTKQNSCTTTTTYHAKQEPREEFSDWPHGLLAIGTFGNNSEIKESQEKQNTCSEDPSSSEEITDFTPEEIGKLQKELTKLLRRKPSVEKEISELPLDRFLNCPSSLEVDRRISNALCSDSGDKEEDIEKTLSVILGKCKDICADNSKKAIGKKSLSFLLKKIFVCRSGFAPTPSLRDTLQESRMEKLLRTILHKKICTQNSSRSPVMKKCIQDKKSATKKRNVEEAEERKDDGCKWVKTDSEYIVLEI |
| AH12G04310 | MKIFNWVHKRFNHKPPKDGFASDMKKNEVTISNDDNNKVVGDNEVALLKQVALTNMLGGWKDGILTIGTLGYDHKEYFGLQVQDKKLLGGDGGLNKNKNNNNNNVFDDYAENDDEEVNPLMLNTFEHHNNNFDDDDDDDEEVHVGGGANQIIIDANYNVMTKEEVLINNEIVLADESNDDDDDGESDDVDDDQKKKIVRGERITLADLFLADSEVKKKIMGPTKILVESNDDEKSNLKVKHGKSFAKKLLPNVKDNPQPMKDIKKLMKKMLKRKIHPDFDAKNQKPEKKESIDGNHTKKKEGRKASIYCIPI |
| AH12G21010 | MQILQWVLFKRVHEQKGECNISDTPTKREATSTTYKKGKGKDIVALKHYSRRYKRTKRVEESKIGCIRLKAFAFICGKDIPKACFYRTLNLNKRRDFVHSMKMKREELSRGVGITKNEDSSSSSASQNVGNKKVLPITEGSSTNSSERSEQHFKTVENKKNKKKSISKMKEILRWAAAAKSDKSRRKFNGQKVLEFRRDGTTKSSSVKNEDDEVGIESPKISFSLDMERCSTTYSVYSSATSTDSYSFIENQRAHIAHSNINVSVQGCGYTNNCRHENWITTDSEFVVLEL |
| AH13G27590 | MKILQWMQKRINGNDEKKKPSPISTTYYMRHYEPCKQEFGDWPQALLAIGTFGTNSDNQKEDSEKTNKGRALDPPCFQDCEDEFTLEEVGSLQNEFNKYFQVPEEPNSAAKPLSTENSSLEEEGHYGDFHQSSSLMDAKGKDQCLDHSRKNGVAKKSLNFLLKKMLVCNSGFQPTTPFLKDPSLSTESRMEKILRAILNKKIYPQGTSSTMFIKKYLETKSIPQYYSDDDDNDDDEFAKAVVNGCKWVKTDAEYIVLEI |
| AH14G41310 | MKLLGWMHRKFRQNNSSEPFKDLVIGNSCNCLSGQSPLDDEQNYLKPNNHGVKLFKNMNQKVMRKSFAGIDSTKEHEDYYIEPSSMCDDDLFPGFLAIGTLGSSSDQVVSDPTSTPTFAISVESITEKEDEVTENDLKLINDELEKVLGADDVSIDYYSSGRNSHVSTGRNSHVSTGRSSHVSIITLSGKPITETHNNNNGGSVCPLQGYLFGTAIELSETTRTSSSSSSTMTAAATATATTKKEHRTSLGELFQRSKLAEECAKNNNENKEEKREGDKSAMNLVKEKLKKRILHHASSRNSTSMNDSSSSAAETKLNKILHMFRKKVHPESSTAAHKYSKQHRKNENKKKIMSDIKGSEIVHPDYDENEDSFINREHWIKTDADYLVLEL |
| AH16G14050 | MKIVNWVHKRFHHSTTLKDGFASNMKNIEPMRSNNEDSEGMMKQVALAELFGGWKDGILTIGTLGCDPLINSYSQNKQYYALESEEDQEEEEEQEDEENYNGEDDNEEVNPLMQSTFEEVKKVDVTDENSIEEMEKKKKGERITLADLFLADSDVKMEGAKSKVSTADEDEKQSSIMKGKHHMHALSFTKKLIPRGLNKDNPHPIQDIKKLIKKMLKRKIHPELLDVKNPKTSDTNDTASLLLI |
| AH19G13150 | MKFLSWMQNKFSGNQGSTKQNSCTTTTTYHAKQEPREEFSDWPHGLLAIGTFGNNSEIKESQENQNTCSEEPSSSEEIADFTPEEIGKLQKELTKLLRRKPSVEKEISELPLDRFLNCPSSLEVDRRISNALCSDSGDKEEDIEKTLSVILGKCKDICADNSKKAIGKKSLSFLLKKIFVCRSGFAPTPSLRDTLQESRMEKLLRTILHKKICTQNSSRSPVMKKCIQDKKSATKKRNMEEPEERKDDGCKWVKTDSEYIVLEI |

Table S2: Primers used for qPCR.

| **Primer name** | **Primer sequence 5'-3'** | **amplification efficiency (%)** |
| --- | --- | --- |
| AhTAC1-1-F | TTCTTCTTCT TCTTCATCGT TAAAG | 93.3 |
| AhTAC1-1-R | CACTTGGTTATGATCACAAAGAATAC |  |
| AhTAC1-2-F | ATCATGAAAGGAAAGCATCATAATC | 96.7 |
| AhTAC1-2-R | TTGGGA TTCTTAACAT CCAGCAGT |  |
| AhTAC1-3-F | CACTTGGTTATGATCACAAAGAATAt | 95.1 |
| AhTAC1-3-R | ATCATCATCA TCATCATCAT CAAAG |  |
| AhTAC1-4-F | AAAACAAAGTAGTATCATGAAAGGA | 91.4 |
| AhTAC1-4-R | TTGGGA TTCTTAACAT CCAGCAGc |  |
| AhLAZY1-1-F | CCGAGCTATTTCATAGGACAAAG | 93.6 |
| AhLAZY1-1-R | CTTAATATTG CAAATGAATT CGCTGG |  |
| AhLAZY1-3-F | ATGAAGTTACTTGGCTGGATGCATA | 90.6 |
| AhLAZY1-3-R | AACCCAGG GAAGAGATCA TCATC |  |
| AhLAZY3-1-F | TCAAGATTGTGAGGATGAATTCACT | 92.3 |
| AhLAZY3-1-R | TAATGCCCTTCTTCTTCT TCCTC |  |
| AhLAZY5-1-F | GGGATTTTGTTCACTCAATGAAGTC | 91.1 |
| AhLAZY5-1-R | TTTTTGAA ATACTCTTCG TCTTGTTG |  |
| AhLAZY5-2-F | AGGGATTTTGTTCACTCAATGAAGAT | 95.3 |
| AhLAZY5-2-R | TTTTTGAAAT ACTCTTCTTCTTGTTC |  |
| AhActin-F | TCTTCCAGCCATCCATGATCGGG | 98.8 |
| AhActin-R | GCTACTCGGTGCCAATGCTGT |  |

Table S3: The Ka, Ks information of IGT genes in *A. hypogaea, A. ipaensis and A. duranenesis.*

| **Gene_Name** | **Gene_Name** | **Ka** | **Ks** | **Ka_Ks** | **Selection pressure** |
| --- | --- | --- | --- | --- | --- |
| Araip.HP7FW.1 | AhTAC1-3 | 0 | 0 | None | Purifying selection |
| Araip.PCU2Z.1 | AhLAZY5-2 | 0.00298 | 0.005437 | 0.547301 | Purifying selection |
| Araip.83J75.1 | AhLAZY3-2 | 0 | 0 | None | Purifying selection |
| Araip.9A27H.1 | AhLAZY1-3 | 0 | 0 | None | Purifying selection |
| Araip.W2R6A.1 | AhTAC1-4 | 0 | 0 | None | Purifying selection |
| Araip.MR79R.1 | AhLAZY2/4-2 | 0 | 0 | None | Purifying selection |
| Aradu.M7LVY.1 | AhTAC1-1 | 0.002885 | 0.006036 | 0.477883 | Purifying selection |
| Aradu.AUZ6Q.1 | AhLAZY5-1 | 0.058004 | 0.089832 | 0.645689 | Purifying selection |
| Aradu.20JPA.1 | AhLAZY1-2 | None | None | None | Purifying selection |
| Aradu.JH1LG.1 | AhLAZY2/4-1 | 0.018046 | 0.036046 | 0.500648 | Purifying selection |

Table S4: GO annotation of AhIGT genes

| **Gene_ID** | **GO_ID** | **Type** | **Go Name** |
| --- | --- | --- | --- |
| AhLAZY1-3 | GO:0010817 | biological_process | regulation of hormone levels |
| AhLAZY1-3 | GO:0005634 | cellular_component | nucleus |
| AhLAZY1-3 | GO:0009926 | biological_process | auxin polar transport |
| AhLAZY1-3 | GO:0005575 | cellular_component | cellular_component |
| AhLAZY1-3 | GO:0044424 | cellular_component | obsolete intracellular part |
| AhLAZY1-3 | GO:0071944 | cellular_component | cell periphery |
| AhLAZY1-3 | GO:2000012 | biological_process | regulation of auxin polar transport |
| AhLAZY1-3 | GO:0110165 | cellular_component | cellular anatomical entity |
| AhLAZY1-3 | GO:0016020 | cellular_component | membrane |
| AhLAZY1-3 | GO:0008150 | biological_process | biological_process |
| AhLAZY1-3 | GO:0043226 | cellular_component | organelle |
| AhLAZY1-3 | GO:0006810 | biological_process | transport |
| AhLAZY1-3 | GO:0043227 | cellular_component | membrane-bounded organelle |
| AhLAZY1-3 | GO:0043229 | cellular_component | intracellular organelle |
| AhLAZY1-3 | GO:0043231 | cellular_component | intracellular membrane-bounded organelle |
| AhLAZY1-3 | GO:0051234 | biological_process | establishment of localization |
| AhLAZY1-3 | GO:0050789 | biological_process | regulation of biological process |
| AhLAZY1-3 | GO:0051049 | biological_process | regulation of transport |
| AhLAZY1-3 | GO:0051179 | biological_process | localization |
| AhLAZY1-3 | GO:0065007 | biological_process | biological regulation |
| AhLAZY1-3 | GO:0032879 | biological_process | regulation of localization |
| AhLAZY1-3 | GO:0065008 | biological_process | regulation of biological quality |
| AhLAZY1-3 | GO:0044464 | cellular_component | obsolete cell part |
| AhLAZY1-3 | GO:0005622 | cellular_component | intracellular anatomical structure |
| AhLAZY1-3 | GO:0060918 | biological_process | auxin transport |
| AhLAZY1-3 | GO:0005623 | cellular_component | obsolete cell |
| AhLAZY1-3 | GO:0009914 | biological_process | hormone transport |
| AhLAZY1-3 | GO:0005886 | cellular_component | plasma membrane |
| AhLAZY1-1 | GO:0005634 | cellular_component | nucleus |
| AhLAZY1-1 | GO:0009605 | biological_process | response to external stimulus |
| AhLAZY1-1 | GO:0009606 | biological_process | tropism |
| AhLAZY1-1 | GO:0009958 | biological_process | positive gravitropism |
| AhLAZY1-1 | GO:0005575 | cellular_component | cellular_component |
| AhLAZY1-1 | GO:0009959 | biological_process | negative gravitropism |
| AhLAZY1-1 | GO:0044424 | cellular_component | obsolete intracellular part |
| AhLAZY1-1 | GO:0071944 | cellular_component | cell periphery |
| AhLAZY1-1 | GO:0050896 | biological_process | response to stimulus |
| AhLAZY1-1 | GO:0044464 | cellular_component | obsolete cell part |
| AhLAZY1-1 | GO:0110165 | cellular_component | cellular anatomical entity |
| AhLAZY1-1 | GO:0016020 | cellular_component | membrane |
| AhLAZY1-1 | GO:0005622 | cellular_component | intracellular anatomical structure |
| AhLAZY1-1 | GO:0008150 | biological_process | biological_process |
| AhLAZY1-1 | GO:0005623 | cellular_component | obsolete cell |
| AhLAZY1-1 | GO:0043226 | cellular_component | organelle |
| AhLAZY1-1 | GO:0043227 | cellular_component | membrane-bounded organelle |
| AhLAZY1-1 | GO:0009628 | biological_process | response to abiotic stimulus |
| AhLAZY1-1 | GO:0043229 | cellular_component | intracellular organelle |
| AhLAZY1-1 | GO:0009629 | biological_process | response to gravity |
| AhLAZY1-1 | GO:0009630 | biological_process | gravitropism |
| AhLAZY1-1 | GO:0005886 | cellular_component | plasma membrane |
| AhLAZY1-1 | GO:0043231 | cellular_component | intracellular membrane-bounded organelle |
| AhLAZY1-2 | GO:0005634 | cellular_component | nucleus |
| AhLAZY1-2 | GO:0009605 | biological_process | response to external stimulus |
| AhLAZY1-2 | GO:0009606 | biological_process | tropism |
| AhLAZY1-2 | GO:0009958 | biological_process | positive gravitropism |
| AhLAZY1-2 | GO:0005575 | cellular_component | cellular_component |
| AhLAZY1-2 | GO:0009959 | biological_process | negative gravitropism |
| AhLAZY1-2 | GO:0044424 | cellular_component | obsolete intracellular part |
| AhLAZY1-2 | GO:0071944 | cellular_component | cell periphery |
| AhLAZY1-2 | GO:0050896 | biological_process | response to stimulus |
| AhLAZY1-2 | GO:0044464 | cellular_component | obsolete cell part |
| AhLAZY1-2 | GO:0110165 | cellular_component | cellular anatomical entity |
| AhLAZY1-2 | GO:0016020 | cellular_component | membrane |
| AhLAZY1-2 | GO:0005622 | cellular_component | intracellular anatomical structure |
| AhLAZY1-2 | GO:0008150 | biological_process | biological_process |
| AhLAZY1-2 | GO:0005623 | cellular_component | obsolete cell |
| AhLAZY1-2 | GO:0043226 | cellular_component | organelle |
| AhLAZY1-2 | GO:0043227 | cellular_component | membrane-bounded organelle |
| AhLAZY1-2 | GO:0009628 | biological_process | response to abiotic stimulus |
| AhLAZY1-2 | GO:0043229 | cellular_component | intracellular organelle |
| AhLAZY1-2 | GO:0009629 | biological_process | response to gravity |
| AhLAZY1-2 | GO:0009630 | biological_process | gravitropism |
| AhLAZY1-2 | GO:0005886 | cellular_component | plasma membrane |
| AhLAZY1-2 | GO:0043231 | cellular_component | intracellular membrane-bounded organelle |

Table S5: Expression information of *AhIGT* genes in various tissues (FPKM).

| **Gene ID** | **Cotyledon** | **Root** | **Stem** | **Leaves** | **Stem.tip** | **Gynophore** | **Florescence** | **Testa** | **Pericarp** | **Embryo** |
| --- | --- | --- | --- | --- | --- | --- | --- | --- | --- | --- |
| AhLAZY1-1 | 0.24 | 0.07 | 0.59 | 0.15 | 0.33 | 0.21 | 0.19 | 0.24 | 0.08 | 0.49 |
| AhLAZY1-2 | 1.28 | 0.52 | 1.59 | 0.87 | 0.53 | 0.62 | 0.49 | 0.62 | 0.54 | 1.39 |
| AhLAZY1-3 | 0.2 | 0 | 19.09 | 14.71 | 9.74 | 1.97 | 3.72 | 0.37 | 0.15 | 0.21 |
| AhLAZY2/4-1 | 0.04 | 0.25 | 1.73 | 0.75 | 3.2 | 0.14 | 0.8 | 0 | 0 | 0.12 |
| AhLAZY2/4-2 | 0.24 | 0.85 | 4.02 | 0.89 | 6.32 | 0.51 | 1.43 | 0.06 | 0.16 | 0.39 |
| AhLAZY3-1 | 0 | 0.1 | 0 | 0.09 | 0.13 | 0.06 | 0.75 | 0.15 | 0 | 0.15 |
| AhLAZY3-2 | 0 | 0 | 0.04 | 0.07 | 0.03 | 0 | 0.19 | 0.09 | 0 | 0 |
| AhLAZY5-1 | 0.22 | 0 | 0.13 | 0.31 | 0.88 | 0 | 0.05 | 0 | 0 | 0 |
| AhLAZY5-2 | 0.13 | 0 | 0.21 | 0.84 | 1.43 | 0.05 | 0.11 | 0.11 | 0 | 0.04 |
| AhTAC1-1 | 0 | 0 | 2.14 | 1.04 | 5.2 | 49.23 | 3.17 | 6.34 | 0.94 | 2.93 |
| AhTAC1-2 | 0.33 | 0.3 | 2.72 | 1.77 | 1.35 | 18.77 | 2.13 | 0.31 | 0.44 | 0.48 |
| AhTAC1-3 | 0 | 0 | 3.04 | 1.42 | 4.88 | 48.23 | 4.14 | 0 | 1.74 | 0.14 |
| AhTAC1-4 | 0.32 | 2.69 | 3.42 | 1.76 | 2.05 | 19.17 | 2.06 | 5.3 | 1.33 | 6.09 |


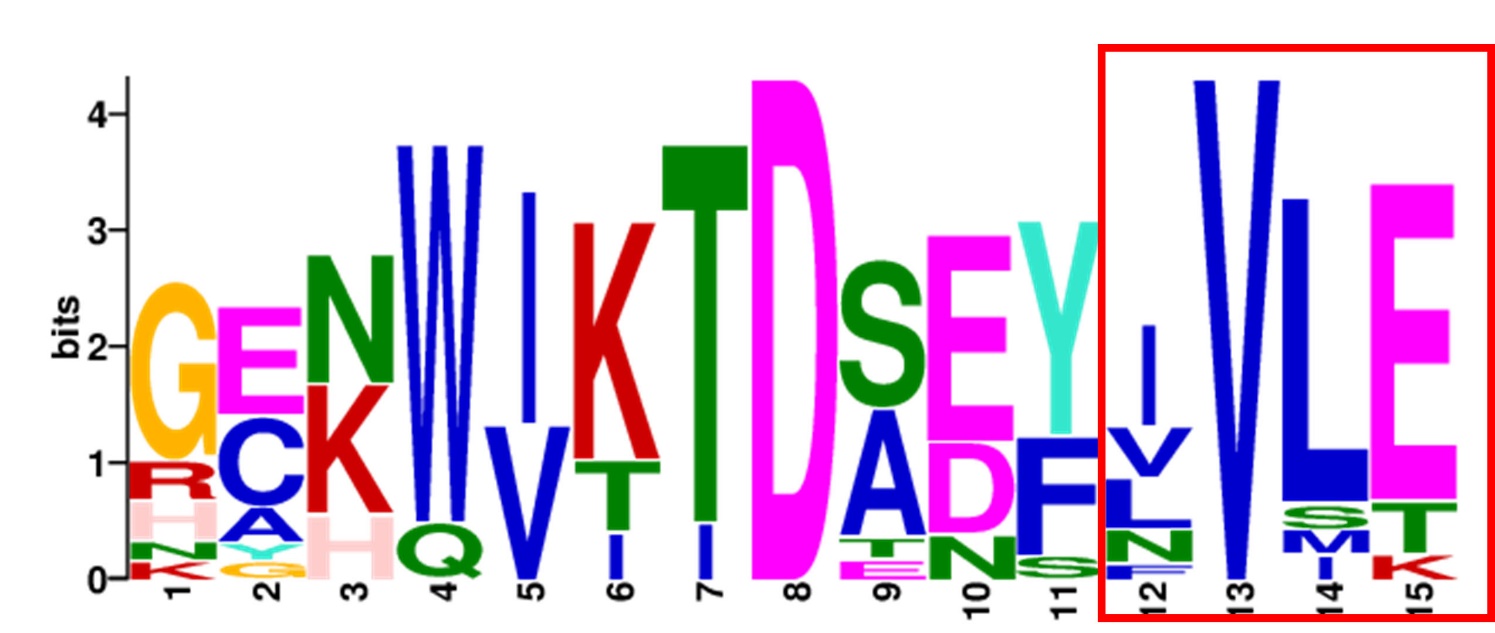


Figure S1: The amino acid sequence of conserved motif 3. Red box indicated EAR motif.
